# Selective lysosomal H_2_O_2_–ROS imaging with a naphthalimide probe forming hydroxylamine without overoxidation

**DOI:** 10.1039/d6ra00730a

**Published:** 2026-05-14

**Authors:** Ricardo Flores-Cruz, Nitzya Ruiz-Robledo, Adriana Romo-Pérez, Arturo Jiménez-Sánchez

**Affiliations:** a Instituto de Química, Universidad Nacional Autónoma de México, Ciudad Universitaria Coyoacán 04510 Mexico City Mexico arturo.jimenez@iquimica.unam.mx

## Abstract

Hydrogen peroxide (H_2_O_2_) plays a central role in oxidative stress, signaling, and pathophysiology, yet its highly dynamic and compartmentalized distribution in living cells makes precise monitoring a major analytical challenge. Here, we present LysoH_2_O_2_, a naphthalimide-derived fluorescent probe specifically designed for lysosomal H_2_O_2_ detection and imaging. The probe exploits a selective transformation of an amine group into a hydroxylamine moiety upon reaction with H_2_O_2_, leading to a pronounced fluorescence enhancement. This molecular design integrates a lysosome-targeting unit to ensure subcellular specificity, enabling real-time visualization of H_2_O_2_ fluctuations in the lysosomal microenvironment. Confocal fluorescence microscopy demonstrates the ability of LysoH_2_O_2_ to track endogenous and exogenous H_2_O_2_ with high sensitivity and minimal cytotoxicity in living cells. Distinct from conventional boronate-based probes, LysoH_2_O_2_ employs a novel, biocompatible chemical transformation to achieve sensitive and selective monitoring of H_2_O_2_ within lysosomes.

## Abstract 2

Hydrogen peroxide (H_2_O_2_) plays a central role in oxidative stress, signaling, and pathophysiology, yet its highly dynamic and compartmentalized distribution in living cells makes precise monitoring a major analytical challenge.

## Graphical abstract

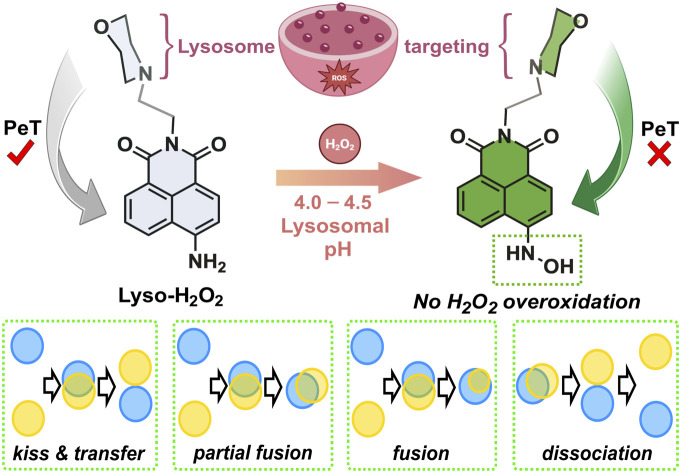

## Introduction

Within the diverse family of reactive oxygen species (ROS), hydrogen peroxide (H_2_O_2_) is uniquely versatile, serving as a essential secondary messenger in healthy cells while driving oxidative damage in pathological states.^1,2^ This dualistic nature implicates H_2_O_2_ in a wide array of functions, from immune defense to cell proliferation, and its dysregulation is a key factor in diseases including cancer, neurodegeneration, and diabetes.^2,3^ Gaining a precise understanding of H_2_O_2_ biological roles therefore depends on our ability to track its production and flux within the complex environment of a living cell, particularly at the subcellular level.^4,5^ Consequently, developing robust and sensitive methods for real-time H_2_O_2_ detection in complex biological environments remains a critical research imperative. Current methodologies for H_2_O_2_ detection, while varied, often encounter limitations including insufficient sensitivity, poor selectivity in complex biological milieus, and an inability to provide real-time, *in situ* monitoring.^6^ The pursuit of this goal has led to the development of various detection strategies. While techniques like electrochemistry and enzymatic assays offer quantification, they generally lack the spatial resolution needed for organelle-specific analysis and are often restricted to extracellular environments.^7^ Chromatographic methods, though highly specific, are inherently incompatible with the real-time, non-invasive imaging of dynamic H_2_O_2_ changes in live cells. Fluorescence microscopy, empowered by synthetic molecular probes, has thus become the method of choice, providing unmatched spatiotemporal resolution for monitoring ROS in their native biological context.^8–10^

Significant progress has been made in designing small-molecule fluorescent probes for H_2_O_2_, with many recent efforts focusing on organelle specificity. A number of effective designs target mitochondria, capitalizing on its membrane potential, and others have been engineered for lysosomes by incorporating pH-sensitive targeting groups like morpholine.^11–19^ Despite these advances, the chemical mechanisms underpinning most H_2_O_2_ probes remain limited. The vast majority rely on the boronate ester recognition unit, which undergoes H_2_O_2_-mediated cleavage to reveal a fluorophore.^11,20^ Moreover, most existing probes rely on the common boronate ester motif, which reacts with H_2_O_2_ to produce the corresponding phenol (or in some cases boric acid diester) alongside quinone byproducts, species known to exhibit cytotoxicity.^21^ In contrast, our work introduces a lysosome-targeted probe that, for the first time, undergoes a direct chemical transformation from an amine to a hydroxylamine (azanol) derivative within the same fluorophore scaffold, without generating harmful byproducts. A detailed reaction mechanism scheme is shown in Fig. S10, SI.^22–24^ This novel reaction not only ensures biocompatibility but also expands the toolkit for selective monitoring of H_2_O_2_.

**Fig. 1:**
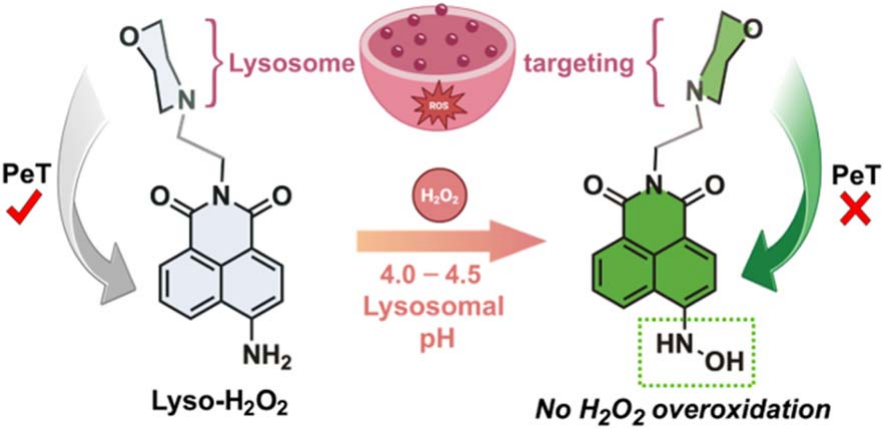
Chemical structure of the LysoH_2_O_2_ probe and proposed sensing mechanism upon lysosome targeting to form a lysosome stabilized hydroxylamine function. The probe was designed to activate the green (GFP, *λ*_exc_ = 488 nm, *λ*_em_ = 530–550 nm) confocal channel, through photoinduced electron transfer (PeT) process inhibition.

This work introduces LysoH_2_O_2_, a fluorescent probe that overcomes limitations of conventional boronate-based designs. It introduces a first-in-class, biocompatible recognition mechanism where H_2_O_2_ directly converts an aromatic amine to a stable hydroxylamine within the naphthalimide scaffold,^25^ avoiding cytotoxic byproducts. This transformation is uniquely stabilized by the acidic lysosomal environment, preventing overoxidation.^26^ By integrating this novel chemistry with lysosomal targeting, LysoH_2_O_2_ enables specific, high-fidelity imaging of redox dynamics in this organelle, Fig. 1.

## Experimental

### Materials and measurements

All chemical reagents and solvents were procured from commercial suppliers (*e.g.*, Aldrich) and used as received, unless stated otherwise. Tetrahydrofuran (THF) was purified by distillation from sodium/benzophenone under an inert argon atmosphere. Reaction progress was monitored by thin-layer chromatography (TLC) using silica gel plates with UV254 indicator, visualized under ultraviolet light at 254 and 365 nm. Nuclear magnetic resonance (NMR) spectra for ^1^H and ^13^C were acquired on Bruker (400 MHz) and Jeol unity (300 MHz) spectrometers at ambient temperature, with chemical shifts (*δ*) reported in parts per million (ppm) relative to tetramethylsilane (TMS) as an internal standard. High-resolution mass spectrometric (HRMS) analysis was conducted with an Agilent Technologies 6530 Accurate-Mass Q-TOF LC/MS system equipped with an electrospray ionization (ESI) source. Photophysical characterization, including fluorescence emission and UV-vis absorption spectra, was performed using an Edinburgh Instruments FS500 spectrofluorometer (or a Cary Eclipse fluorimeter) and a Thermo Scientific Evolution diode array spectrophotometer, respectively.

### Cell culture, confocal microscopy and imaging analysis

Human pulmonary adenocarcinoma epithelial cells (SK-Lu-1, ATCC HTB-57) were maintained in RPMI-1640 medium, supplemented with 10% (v/v) fetal bovine serum (FBS), 2 µM l-glutamine, 100 U per mL penicillin G, and 100 µg per mL streptomycin sulfate, in a humidified incubator at 37 °C with 5% CO_2_. For live-cell imaging experiments, cells were seeded into 8-well µ-Slides (ibidi, Germany) at a density of 20 000 cells per well and allowed to adhere for 24 hours in MEM Alpha medium containing 10% FBS. Prior to treatment, the cell culture medium was replaced with serum-free MEM Alpha. The cells were then incubated with LysoH_2_O_2_ probe at specified concentrations (1–5 µM) for varying durations, as detailed for individual experiments. For colocalization studies, LysoTracker™ Deep Red (Thermo Fisher, 50 nM) was applied to the cells for 10 minutes before the addition of LysoH_2_O_2_, following the manufacturer's protocol. To monitor cellular blebs, a separate set of cells was stained with 1 µM AztecBleb^TxR^^27^ for 30 minutes. Following all staining procedures, cells were rinsed twice with pre-warmed, serum-free MEM Alpha before imaging.

Confocal microscopy was performed using either an inverted Zeiss LSM 880 or a Nikon A1R microscope, both equipped with on-stage environmental chambers to maintain conditions at 37 °C and 5% CO_2_ throughout the imaging sessions. To mitigate potential laser-induced artefacts and autofluorescence, laser power was minimized to 0.05 mW, and background signals from untreated control cells were recorded and digitally subtracted from experimental images. For time-course experiments, cells within the microscope chamber were imaged at 5 minute intervals over a period of up to 4 hours. Fluorescence images were acquired using consistent settings for all samples within a given experiment. Co-staining protocols were employed to validate the subcellular localization of the probe. For quantitative analysis, regions of interest (ROIs) corresponding to individual lysosomes were manually delineated in at least 40 cells per condition using the freehand selection tools in Fiji/ImageJ software. Data from three independent biological replicates were pooled, and results are expressed as the mean ± standard error of the mean (s.e.m.). Statistical analyses were performed using GraphPad Prism software to assess the significance of observed differences.

### Compounds synthesis and chemical characterization

The target probe LysoH_2_O_2_, 6-(2-morpholinoethyl)-1*H*-benzo[de]isoquinoline-1,3(2*H*)-dione, was synthesized according to the following procedure. A solution of 4-bromo-1,8-naphthalic anhydride (1 mmol, 0.278 g) and sodium azide (NaN_3_, 3 mmol, 0.195 g) in a mixture of *N*,*N*-dimethylformamide (DMF) and water (20 mL, 10 : 1 v/v) was stirred at 80 °C for 4 hours. Upon completion, the mixture was poured into ice-cold water (10 mL) to induce precipitation. The resulting solid was collected by filtration and subsequently dissolved in anhydrous DMF (20 mL). To this solution, aqueous sodium hydrosulfide (NaSH, 4 mmol, 0.224 g in 2 mL H_2_O) was added, and the reaction was stirred at 90 °C for 1 hour. The mixture was then quenched with ice water and acidified to weak acidity, yielding a precipitate that was isolated *via* filtration. This intermediate was combined with 4-(2-aminoethyl)morpholine (1.3 mmol, 0.75 mL) in DMF (10 mL) and heated to 120 °C for 8 hours under an argon atmosphere with vigorous stirring. After cooling to room temperature, the crude product was taken up in acetone (∼25 mL) and purified by flash column chromatography over silica gel, using a gradient of dichloromethane and methanol (95 : 5) as the eluent. LysoH_2_O_2_ was obtained as a yellow solid in a 67% yield (0.225 g). ^1^H NMR (300 MHz, CDCl_3_) *δ*: 8.61 (dd, *J* = 26.60 Hz, 1H), 8.42 (dd, *J* = 12.87 Hz, 1H), 8.12 (dd, *J* = 21.45 Hz, 1H), 7.68 (m, *J* = 22.32 Hz, 1H), 6.90 (d, *J* = 13.73 Hz, 1H), 4.97 (s, 2H), 4.34 (t, *J* = 21.45 Hz, 2H), 3.71 (m, *J* = 21.45 Hz, 4H), 2.72 (t, *J* = 26.60 Hz, 2H), 2.63 (s, 4H). ^13^C NMR (75 MHz, CDCl_3_) *δ*: 154.2, 147.1, 133.7, 129.3, 127.2, 124.8, 122.1, 119.5, 117.21, 109.4, 107.81, 67.25, 57.16, 55.18, 36.41. HRMS (ESI^+^) *m*/*z*: calculated for C_18_H_20_N_3_O_3_ [M + H]^+^: 326.14. Found: 326.1498.

**Fig. 2:**
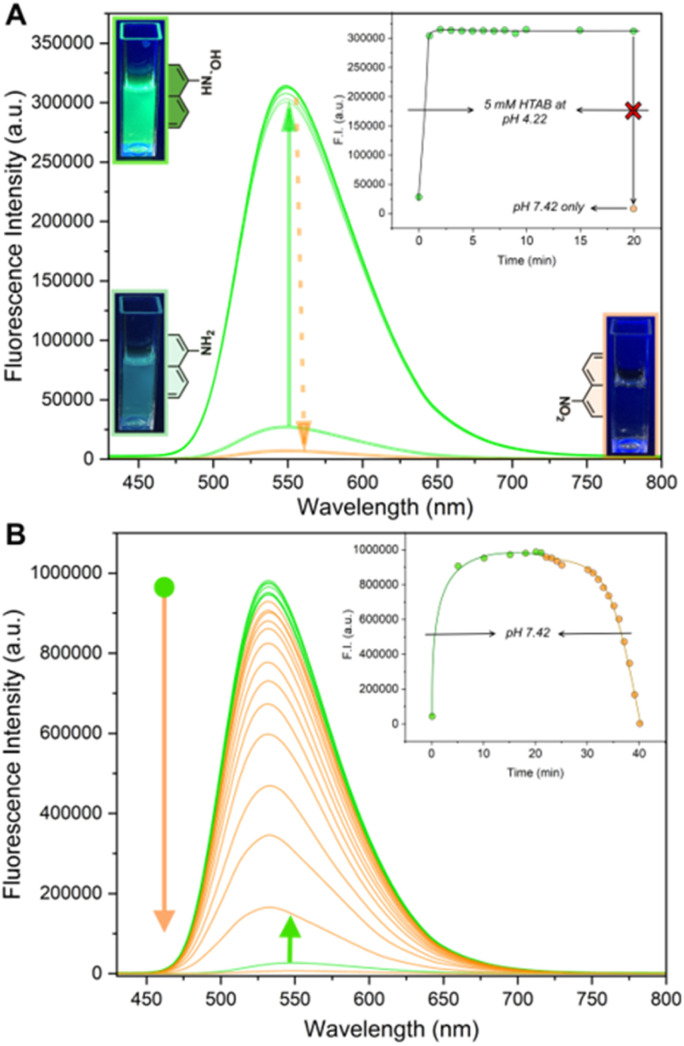
Fluorescence titration profiles (*λ*_exc_ = 430 nm) of 40 µM LysoH_2_O_2_ probe under: (A) exposure to 5 equiv. H_2_O_2_ at pH 4.22 and 5 mM HTAB micellar medium, exhibiting a strong fluorescence activation at 550 nm, following oxidation to the hydroxylamine intermediate it displays a stable fluorescence, confirming resistance to further oxidation under mildly acidic conditions. (B) In contrast, in pure aqueous media at pH 7.42, overoxidation to the nitro derivative occurs, leading to complete fluorescence quenching. Insets show fluorescence *vs.* H_2_O_2_ time-course profiles at selected wavelengths. Arrows show directions of the spectral changes on time.

The ^1^H NMR spectrum of the hydroxylamine product (Fig. S5) displays two characteristic exchangeable singlets in the downfield region. The broader signal at *δ* 10.15 ppm is attributed to the –OH proton, whose increased linewidth reflects the faster exchange kinetics typical of hydroxyl protons; D_2_O addition caused preferential attenuation of this signal over the *δ* 11.05 –NH– resonance, further supporting this assignment (data not shown). The more downfield signal at *δ* 11.05 ppm is assigned to the –NH– proton, whose exceptional deshielding is attributed to intramolecular hydrogen bonding between the N–H and the flanking carbonyl oxygens of the naphthalimide scaffold, consistent with literature values for related arylhydroxylamines.^26^ The stability of this product is strongly pH-dependent: under mildly acidic conditions mimicking the lysosomal environment (pH 4.22, 5 mM HTAB), the hydroxylamine signal remains stable over time, as confirmed by the time-course fluorescence profiles in Fig. 2A. In contrast, at physiological pH (7.42), the free-base form of the hydroxylamine undergoes progressive overoxidation to the nitro derivative with concomitant fluorescence quenching, consistent with the acid-stabilization mechanism described in Fig. 2B.

## Results and discussion

### Spectroscopic features of the LysoH_2_O_2_ probe

The fluorescence titration profiles of the LysoH_2_O_2_ probe provide compelling evidence supporting the proposed activation mechanism and its pronounced dependence on the surrounding microenvironment.

The excitation and emission spectral profiles of LysoH_2_O_2_ before and after H_2_O_2_ activation at pH 4.22 and pH 7.42 are presented in Fig. S11 (SI), illustrating the pronounced fluorescence enhancement at lysosomal pH and the contrasting signal quenching at neutral pH due to overoxidation.

Quantitative photophysical characterization in 5 mM HTAB micellar medium at pH 4.22 revealed a molar extinction coefficient of *ε* = 6550 M^−1^ cm^−1^ and a fluorescence quantum yield of *Φ* = 0.008 for LysoH_2_O_2_, increasing to *ε* = 6890 M^−1^ cm^−1^ and *Φ* = 0.21 for the hydroxylamine product upon H_2_O_2_ activation, corresponding to a 26.3-fold fluorescence enhancement consistent with the PeT inhibition mechanism (Table S1, SI). Initially, the probe exhibits a weak fluorescence signal at 550 nm; however, upon exposure to H_2_O_2_, a marked enhancement of fluorescence intensity is observed at this wavelength. This activation arises from the inhibition of the photoinduced electron transfer (PeT) process from the amino group to the naphthalimide fluorophore following its oxidative transformation. To further support the PeT-based turn-on mechanism, a frontier orbital energy alignment diagram was constructed comparing LysoH_2_O_2_ and its hydroxylamine product at the PBE/6-31G(d,p) level of theory (Fig. S12, SI). NBO analysis confirms significant nitrogen lone pair character in the HOMO of the intact probe (HOMO = −5.85 eV), consistent with an active donor-to-acceptor PeT process that quenches fluorescence. Upon oxidation to the hydroxylamine, the lone pair contribution to the HOMO is markedly reduced (HOMO = −5.92 eV) and the HOMO–LUMO gap widens from 2.25 to 2.41 eV (ΔΔ*E* = +0.16 eV), confirming that oxidation lowers the donor orbital energy, suppresses PeT, and restores fluorescence emission.

**Fig. 3:**
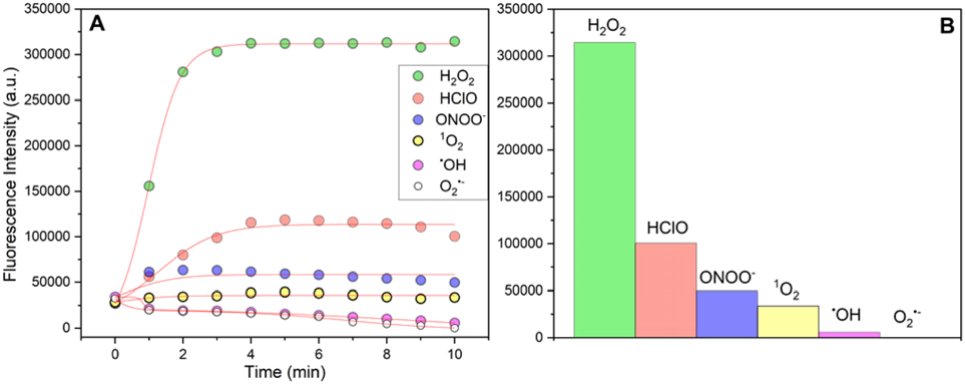
(A) Fluorescence time profiles (*λ*_exc_ = 430 nm, *λ*_em_ = 550 nm) of 40 µM LysoH_2_O_2_ probe upon 5 equiv. H_2_O_2_ and various ROS at pH 4.22 and 5 mM HTAB micellar medium. (B) Corresponding bar plot illustrating fluorescence intensity variations.

Such a PeT-based mechanism has been well-documented for related naphthalimide systems and rationalizes the observed fluorescence “turn-on” behavior, Fig. 2. Notably, the probe selectively responds to H_2_O_2_, as no significant fluorescence activation occurs with other biologically relevant reactive oxygen species (ROS) tested, including HOCl, ONOO^−^, ˙OH, ^1^O_2_, and O_2_˙^−^. Instead, only radical species induced partial quenching of fluorescence, which is chemically anticipated if radical additions interact with the probe. Importantly, this quenching does not significantly interfere with the desired *in vitro* monitoring of H_2_O_2_, as shown in Fig. 3.

Once maximum fluorescence emission is attained through the formation of the hydroxylamine intermediate, the signal remains stable under conditions mimicking the lysosomal environment, specifically in 5 mM HTAB micellar medium at pH 4.22. This stability represents a crucial finding, as it confirms that under mildly acidic conditions the hydroxylamine species is preserved, thereby preventing further oxidation and maintaining a consistent fluorescent response. In contrast, when the probe is incubated in pure aqueous media at physiological pH (7.42), overoxidation occurs, leading to the formation of the corresponding nitro derivative and a complete loss of fluorescence. Extended time-course experiments comparing the fluorescence stability of the activated probe at pH 4.22 and pH 7.42 (Fig. S6, SI) revealed a striking contrast: at lysosomal pH, a stable plateau lasting ∼44 minutes precedes a slow pseudo-first-order decay (*k*_obs_ = 0.0076 min^−1^, *t*_1/2_ = 91 min, *R*^2^ = 0.996), with 70.2% of the maximum signal retained at 90 minutes. At neutral pH, overoxidation follows sigmoidal kinetics with a 4.4-fold higher decay rate constant, resulting in near-complete signal quenching (99.4% loss) within the same timeframe. These kinetic data quantitatively establish both the robust stability of the hydroxylamine product under lysosomal conditions and the efficiency of the acid-protection mechanism. This pronounced difference highlights the protective role of the acidic lysosomal environment in stabilizing the fluorescent product and sustaining signal intensity, underscoring the importance of local environmental factors in modulating the probe photophysical response. Importantly, the oxidized product of the hydroxylamine derivative was chemically characterized by ESI-HRMS and ^1^H NMR spectroscopy, confirming the presence of the –NH–OH functional group, Fig. S7–S9, SI file.

These features make LysoH_2_O_2_ highly adaptable for incorporation into subcellular targeting groups with enhanced selectivity and imaging depth. The probe design successfully leverages the unique lysosomal pH to gate the oxidation reaction, providing a selective and stable response to H_2_O_2_ while avoiding the signal loss associated with overoxidation in neutral environments.

### Intracellular localization and lysosomal specificity of LysoH_2_O_2_

Having established the probe mechanism and stability *in vitro*, we next assessed its functionality within living cells. Our primary goals were to confirm its accurate targeting to lysosomes, verify its ability to respond to H_2_O_2_, and demonstrate its application in tracking redox changes during physiological processes like autophagy. The cell viability assessment by MTT assay confirmed that LysoH_2_O_2_ exhibits ∼80% cell viability (∼20% cytotoxicity) across the working concentration range of 1–5 µM in SK-Lu-1 cells, establishing its biocompatibility for live-cell fluorescence imaging applications (Fig. S1, SI).

Confocal microscopy imaging validated the successful lysosomal targeting of LysoH_2_O_2_. While the probe's green channel fluorescence was faint under standard settings, we achieved robust signal detection in the blue channel (DAPI, *λ*_exc_ = 440 nm, *λ*_em_ = 480–500 nm) by optimizing laser power and detector gain. Under these conditions, LysoH_2_O_2_ efficiently entered SK-Lu-1 cells and accumulated in discrete cytoplasmic structures, producing a bright, punctate staining pattern characteristic of lysosomes.

**Fig. 4:**
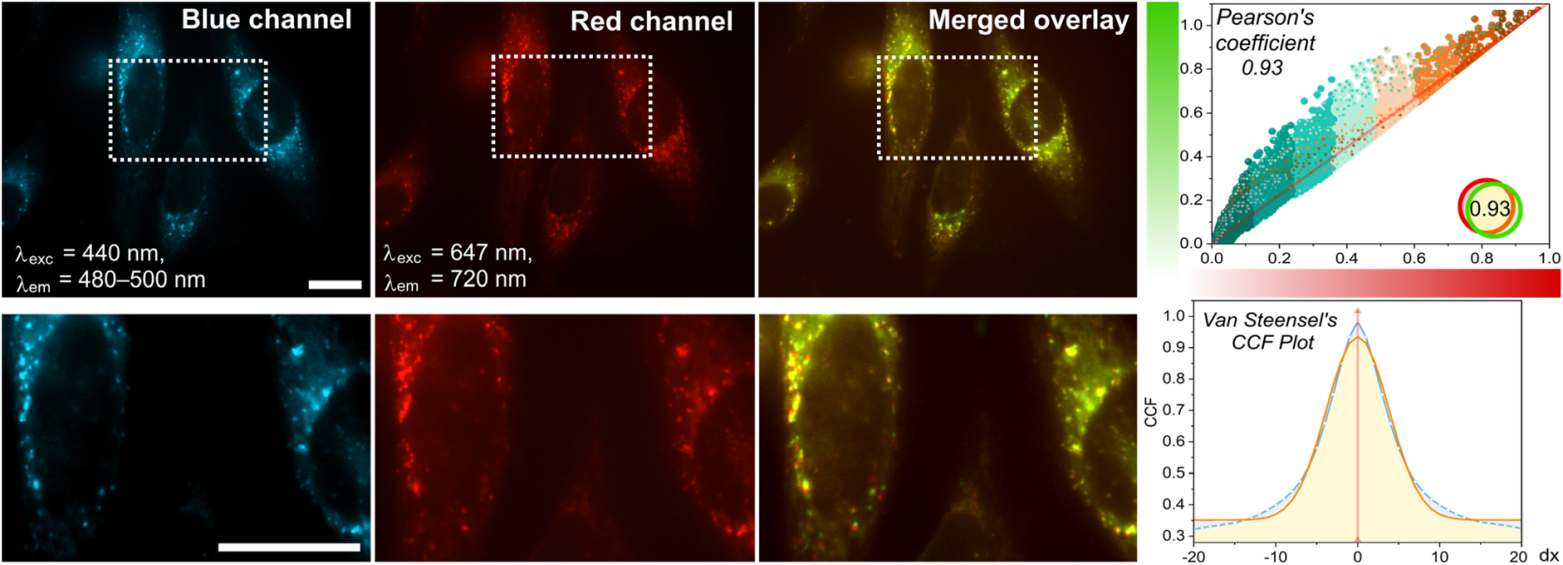
Subcellular colocalization in lysosomes of 5 µM LysoH_2_O_2_ (blue, DAPI: *λ*_exc_ = 440 nm, *λ*_em_ = 480–500 nm) using LysoTracker Deep Red (red, Cy5: *λ*_exc_ = 647 nm, *λ*_em_ = 720) in SK-Lu-1 cells. Right panels display the corresponding Pearson's correlation coefficient (PC > 0.93) and van Steensel's cross-correlation function (CCF) plots, providing quantitative assessment of spatial correlation between fluorescence channels, indicative of its high targeting selectivity. Colocalization is shown as a false-color overlay (green-red overlap rendered in yellow). Scale bars = 20 µm.

To definitively confirm this subcellular localization, we performed co-staining experiments with the established lysosomal marker, LysoTracker™ Deep Red. The resulting fluorescence images showed a striking overlap between the blue signal from LysoH_2_O_2_ and the red signal from LysoTracker (Fig. 4). This visual correlation was strongly supported by quantitative analysis, which yielded a high Pearson's coefficient of 0.935. Further confirmation came from the van Steensel's cross-correlation function (CCF), which displayed a pronounced peak near zero, indicating tight spatial registration of the two signals. Together, these data provide conclusive evidence that the morpholine unit effectively guides LysoH_2_O_2_ to the acidic lysosomal environment, fulfilling the essential requirement of subcellular specificity for subsequent H_2_O_2_ sensing applications.

**Fig. 5:**
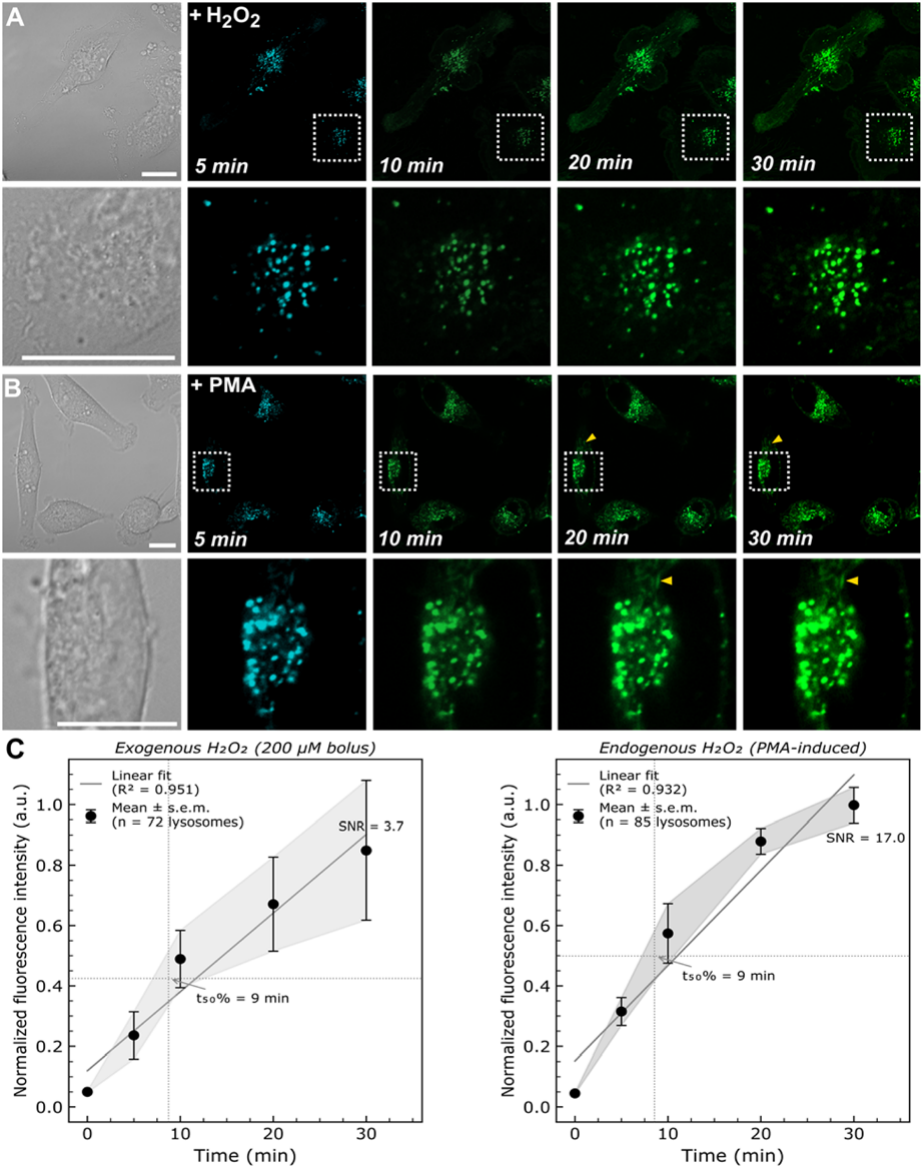
The LysoH_2_O_2_ (7 µM) probe detects H_2_O_2_ from exogenous and endogenous sources in live cells. (A) Fluorescence response to a 200 µM bolus of exogenous H_2_O_2_ starting in the blue (DAPI: *λ*_exc_ = 440 nm, *λ*_em_ = 480–500 nm) and then in the green (GFP: *λ*_exc_ = 488 nm, *λ*_em_ = 510–550 nm) channel. (B) Response to endogenous H_2_O_2_ production induced by PMA, showing signal in lysosomal puncta and subtle response in mitochondria (yellow arrowheads). Images from left to right: brightfield, initial blue channel, and green channel time series showing signal increase. (C) Quantitative mean normalized fluorescence intensity profiles (±s.e.m.) as a function of time for exogenous H_2_O_2_ (left, *n* = 72 lysosomes) and PMA stimulation (right, *n* = 85 lysosomes), extracted from confocal time-lapse images by ImageJ ROI analysis. Arrow indicates time of stimulus addition (*t* = 0). Scale bars = 20 µm.

We next challenged the probe with physiologically relevant H_2_O_2_ stimuli. Treatment of cells with a bolus of exogenous H_2_O_2_ (200 µM) resulted in a rapid and marked activation with an optimal emission in the green fluorescence channel (*λ*_exc_ = 488 nm, *λ*_em_ = 530–550 nm), confirming the ability of the probe to respond to elevated peroxide levels within the cellular context while maintaining a stable fluorescence signal for at least 30 minutes (Fig. 5A). The switch from blue to green channel detection upon H_2_O_2_ stimulation directly reflects PeT inhibition: the intact probe exhibits residual emission in the blue channel (*λ*_exc_ = 440 nm, *λ*_em_ = 480–500 nm), while the hydroxylamine product displays a red-shifted, strongly enhanced emission optimally detected in the green channel (*λ*_exc_ = 488 nm, *λ*_em_ = 510–550 nm).

More significantly, stimulation with phorbol 12-myristate 13-acetate (PMA), a known inducer of endogenous H_2_O_2_ production primarily *via* the NADPH oxidase (NOX) system,^28^ elicited a comparable fluorescent “turn-on” response within lysosomal puncta (Fig. 5B). Interestingly, endogenous ROS production induced by PMA also generated a subtle signal in the green channel at mitochondrial locations (Fig. 5B, yellow arrowheads), which was absent upon direct exogenous H_2_O_2_ addition (Fig. 5A). Several explanations may account for this observation: (i) passive diffusion of NOX-derived H_2_O_2_ from its site of production to neighboring mitochondria; (ii) a minor fraction of the probe residing in mitochondria, rendering it sensitive to locally generated ROS; or (iii) interorganelle ROS communication downstream of NOX activation. Distinguishing between these possibilities will require further investigation using, for example, mitochondria-specific ROS scavengers or dual-organelle co-imaging strategies, and represents a compelling direction for future work.

A key aspect of the LysoH_2_O_2_ probe performance is its specific response to H_2_O_2_ amidst the complex redox landscape of the lysosome. While other reactive species, such as the hydroxyl radical (˙OH), may be present and could potentially quench the formed fluorophore, it is crucial to note that these species do not initiate the fluorescence turn-on. The activation mechanism is specifically gated by the H_2_O_2_-mediated oxidation of the hydroxylamine, a reaction that other ROS/RNS do not trigger under these conditions. Therefore, the observed fluorescence enhancement can be confidently attributed to H_2_O_2_, establishing the probe high operational specificity in a live-cell setting.

The differential response observed between exogenous H_2_O_2_ addition and endogenous PMA stimulation is particularly revealing. The appearance of a mitochondrial signal specifically during endogenous ROS production suggests that the native signaling networks of the cells are being activated. PMA-induced NOX activity likely creates a localized redox signal that is propagated to the mitochondria, potentially as part of a coordinated interorganelle communication event, such as redox relay or calcium signaling. This phenomenon would be entirely masked by the direct application of exogenous H_2_O_2_. The ability of LysoH_2_O_2_ to capture this nuanced difference underscores its unique value; it is not merely a passive reporter of H_2_O_2_ concentration, but an active tool for visualizing compartment-specific ROS signaling cascades in real time. This positions LysoH_2_O_2_ to investigate fundamental questions about how redox signals are generated, contained, and transmitted between organelles.

**Fig. 6:**
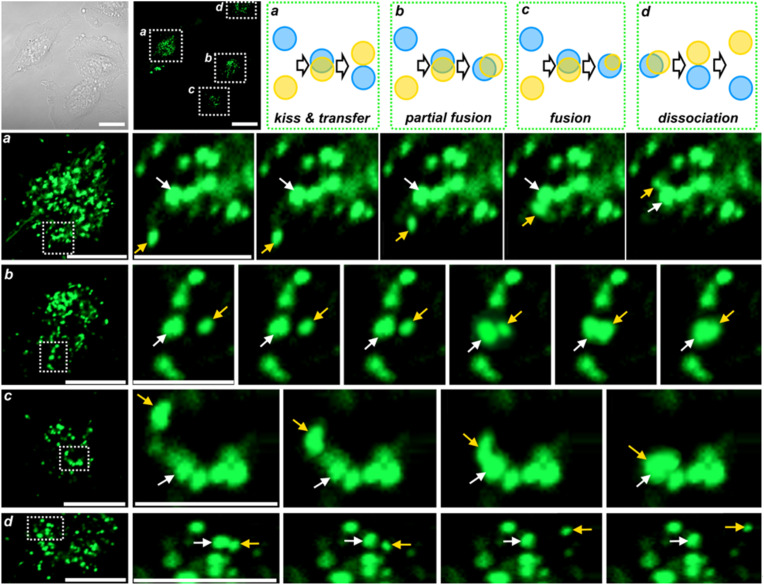
LysoH_2_O_2_ detects a dynamic increase in lysosomal H_2_O_2_ flux during starvation-induced autophagy. Above: full-field confocal microscopy images of SK-Lu-1 cells treated with HBSS buffer to induce autophagy at bright field and green (green, GFP: *λ*_exc_ = 488 nm, *λ*_em_ = 510–550 nm). The white boxes indicate the region shown in the time-lapse series below (a–d). Below: higher-magnification time-lapse series (4 minute intervals) of the boxed region in (a–d), showing LysoH_2_O_2_ fluorescence in individual lysosomes. The panels illustrate dynamic lysosomal processes, including kiss-and-transfer, partial fusion, fusion, and dissociation, illustrated above. To minimize photobleaching, the confocal laser was fully shielded between recordings. Arrows show directions pf lysosomes movement. Scale bars = 20 µm.

Finally, to demonstrate the probe practical utility in cell biology, we employed LysoH_2_O_2_ to monitor H_2_O_2_ dynamics during autophagy in SK-Lu-1 cells. Autophagy was induced using both starvation (HBSS buffer) and the chemical agent rapamycin.^29,30^ Time-lapse confocal microscopy revealed that autophagy induction led to a clear increase in H_2_O_2_-specific fluorescence within newly formed fluorescent puncta, consistent with autophagic vesicles such as autophagosomes and autolysosomes (Fig. 6). We note that the molecular identity of these structures as bona fide autophagosomes was not confirmed by co-labeling with autophagosome-specific markers (*e.g.*, LC3); their characterization as autophagic vesicles is based on their morphology, their dynamic lysosomal fusion and fission behaviors, and the well-established association between starvation-induced autophagy and lysosomal ROS generation.^29^ Co-immunofluorescence confirmation using LC3 reporters is planned for future work. Then, the dynamic interactions between lysosomes during the autophagy process were visualized with the Lyso-H_2_O_2_ probe. As shown in Fig. 6, confocal time-lapse imaging of cells incubated in serum-free medium revealed a sequence of lysosomal fusion and fission events over 4 minutes time-lapse recordings. In Fig. 6a, two lysosomes were observed undergoing a characteristic “kiss-and-transfer” event, in which they gradually approached, transiently interacted to exchange content, and then separated. In Fig. 6b, a partial fusion event was captured, where the lysosomes established a stable contact zone without complete merging. Fig. 6c depicts a full fusion event, where two lysosomes completely merged into a single organelle while maintaining independent motility relative to surrounding structures. Finally, in Fig. 6d, we documented a rare dissociation event, in which a fused lysosome pair progressively separated until complete disengagement and spatial separation were achieved. This result successfully captures a dynamic, physiologically relevant increase in lysosomal H_2_O_2_ flux, underscoring the probe sensitivity and its potential to uncover the role of reactive oxygen species in fundamental cellular processes. However, the present study does not include a pharmacological negative control using autophagy inhibitors (*e.g.*, bafilomycin A1 or 3-methyladenine), which would formally exclude a general stress response as the origin of the observed fluorescence enhancement. This important control is proposed as a priority for future work employing LysoH_2_O_2_ in autophagy-related investigations.

## Conclusions

This study introduces Lyso-H_2_O_2_, a naphthalimide-based fluorescent probe engineered for the selective detection of hydrogen peroxide within lysosomes. The core innovation of this probe lies in its unique sensing mechanism: a H_2_O_2_-triggered conversion of an amine to a hydroxylamine, which is strategically stabilized by the organelle's native acidic pH to prevent overoxidation and ensure a reliable, turn-on fluorescent response. Confocal microscopy studies in live cells verified the probe excellent lysosomal specificity and its capability to track H_2_O_2_ fluxes from both external stimuli and internal cellular processes, such as autophagy.

More broadly, Lyso-H_2_O_2_ addresses a significant methodological gap in redox biology by shifting the focus from the extensively studied mitochondria to the critically important, yet less explored, lysosomal compartment. This tool provides a new chemical means to challenge the prevailing mitochondrial-centric view of cellular oxidative signaling. We anticipate that Lyso-H_2_O_2_ will serve as a foundational tool for future investigations, enabling multiplexed assays that simultaneously monitor ROS across different organelles. Such approaches are crucial for deciphering the complex landscape of interorganelle redox communication and its profound implications for cellular physiology and disease mechanisms.

## Author contributions

Ricardo Flores-Cruz: methodology, imaging microscopy. Nitzya Ruiz-Robledo: synthesis and chemical characterization. Adriana Romo-Pérez: cell culture, imaging experiments. Arturo Jiménez-Sánchez: data analysis, writing-original draft, funding acquisition.

## Conflicts of interest

The authors declare no competing interests.

## Supplementary Material

RA-016-D6RA00730A-s001

## Data Availability

All data supporting the findings of this study are available within the article and its supplementary information (SI). No new code or software was generated during this study. Raw data files are available from the corresponding author upon reasonable request. Supplementary information: original spectroscopic data (NMR, HRMS, UV-vis, and fluorescence spectra) and additional experimental details. See DOI: https://doi.org/10.1039/d6ra00730a.
