# Supplementary material for: Selective lysosomal H2O2–ROS imaging with a naphthalimide probe forming hydroxylamine without overoxidation
Source: RSC Adv. 2026 May 14;16(28):25495–502. doi: 10.1039/d6ra00730a (PMC13182523; doi:10.1039/d6ra00730a)
Supplement: RA-016-D6RA00730A-s001 [file RA-016-D6RA00730A-s001.pdf]

Supplementary Information (SI) for RSC Advances.
This journal is © The Royal Society of Chemistry 2026

Electronic Supplementary Information

Selective Lysosomal H_2O_2 – ROS Imaging with a Naphthalimide Probe Forming Hydroxylamine without Overoxidation

Ricardo Flores-Cruz, Nitzya Ruiz-Robledo,[a] Adriana Romo-Pérez,[a] and Arturo Jiménez-Sánchez*[a]

[a] Instituto de Química, Universidad Nacional Autónoma de México, México D.F. No. 04510, México. * arturo.jimenez@iquimica.unam.mx

METHODS

Cell Viability Assay (MTT Assay) in SK-Lu-1 Cell Line

Cell viability was evaluated using the colorimetric MTT assay, as originally described by Mosmann (1983), with minor modifications. Briefly, cells were seeded in 96-well plates at a density of 5×10^3 to 1×10^4 cells per well, depending on the cell line, in a final volume of 100 µL of appropriate culture medium. Cell viability was assessed using the MTT (3-(4,5-dimethylthiazol-2-yl)-2,5-diphenyltetrazolium bromide) colorimetric assay in SK-Lu-1 cells treated with **LysoH$_2$O$_2$** across a concentration gradient of 1–5 µM for 24 hours.

At the end of the treatment period, 10 µL of MTT solution (5 mg/mL in phosphate-buffered saline, PBS) was added to each well and the plates were incubated for an additional 3–4 hours at 37 °C under a humidified 5% CO_2 atmosphere. During this incubation, metabolically active cells reduced the yellow tetrazolium salt (MTT) to insoluble purple formazan crystals.

Following incubation, the medium was carefully removed and 100 µL of a solubilization solution (typically dimethyl sulfoxide, DMSO) was added to each well to dissolve the formazan crystals. Absorbance was measured at 570 nm using a microplate reader, with background subtraction at 630–690 nm where applicable. All treatments were performed in triplicate or quadruplicate, and data were expressed as percentage of viable cells relative to untreated controls. Control experiments were performed using LysoTracker™ Deep Red (Invitrogen, L12492) to validate lysosomal targeting and staining specificity. Cell viability assessment by MTT assay confirmed that **LysoH$_2$O$_2$** exhibits ~80% cell viability (~20% cytotoxicity) across the working concentration range of 1–5 µM in SK-Lu-1 cells, establishing its biocompatibility for live-cell fluorescence imaging applications.

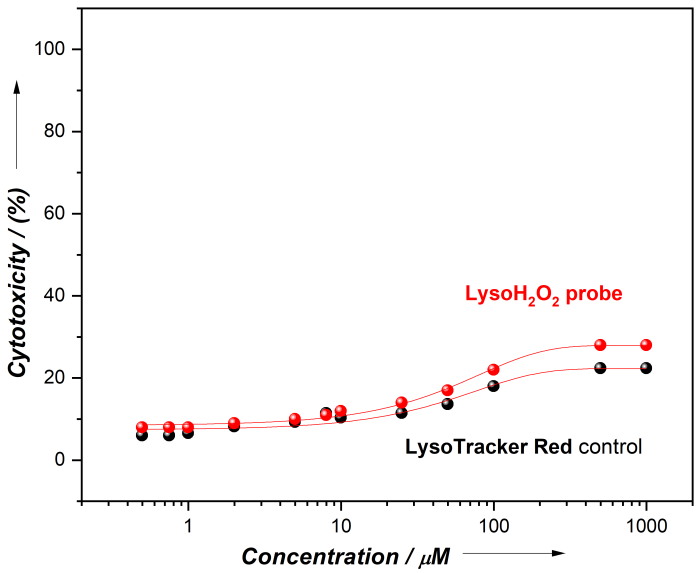

Figure S1. Cell viability assessment by MTT assay confirmed that **LysoH_2O_2** exhibits ~80% cell viability (~20% cytotoxicity) across the working concentration range of 1–5 μM in SK-Lu-1 cells, establishing its biocompatibility for live-cell fluorescence imaging applications.

General Probe Synthesis

Synthesis of 6-(2-morpholinoethyl)-1*H*-benzo[*de*]isoquinoline-1,3(2*H*)-dione (**LysoH_2O_2**). General Methods: All commercial reagents were used as received. NMR spectra were recorded on a 300 MHz spectrometer. Chemical shifts (δ) are reported in ppm relative to residual solvent peaks. High-resolution mass spectrometry (HRMS) was performed using electrospray ionization (ESI).

Procedure: A mixture of 4-bromo-1, 8-naphthalic anhydride, 1 mmol, 0.278 g and sodium azide NaN_3 (0.195 g, 3 mmol) were dissolved in *N,N*-dimethylformamide (DMF) / H_2O (v/v, 10/1, 20 mL) and stirred at 80 °C for 4 h. Then, the solvent was poured into ice water (10 ml), and the precipitated solid was filtered.Then, the solid was dissolved in DMF (20 ml), added with 2ml of aquous NaHS (0.224 g, 4 mmol) and further stirred at 90 °C for 1h. Next, the reaction solution was added with ice water and adjusted to weak acidity. Subsequently, a solid was filtered and added 10 mL DMF containing 4-(2-aminoethyl)morpholine (1.3 mmol, 0.75 mL) and heated at 120 °C under controlled pressure for 8 hours with vigorous stirring. After cooling to room temperature, the crude material was dissolved in acetone (~25 mL) and purified by flash column chromatography on silica gel (eluent: DCM/MeOH 95:5). The product was isolated as a yellow solid. Yield: 67% (0.225 g). ^{1}H NMR (300 MHz, $CDCl_3$) δ: 8.61 (dd, J = 26.60 Hz, 1H), 8.42 (dd, J = 12.87 Hz, 1H), 8.12 (dd, J = 21.45 Hz, 1H), 7.68 (m, J = 22.32 Hz, 1H), 6.90 (d, J = 13.73 Hz, 1H), 4.97 (s, 2H), 4.34 (t, J = 21.45 Hz, 2H), 3.71 (m, J = 21.45 Hz, 4H), 2.72 (t, J = 26.60 Hz, 2H), 2.63 (s, 4H). ^{13}C NMR (75 MHz, $CDCl_3$) δ 154.2, 147.1, 133.7, 129.3, 127.2, 124.8, 122.1, 119.5, 117.21, 109.4, 107.81, 67.25, 57.16, 55.18, 36.41. HRMS (ESI+) m/z: Calculated for $C_{18}H_{20}N_3O_3$ $[M+H]^+$: 326.14. Found: 326.1498.

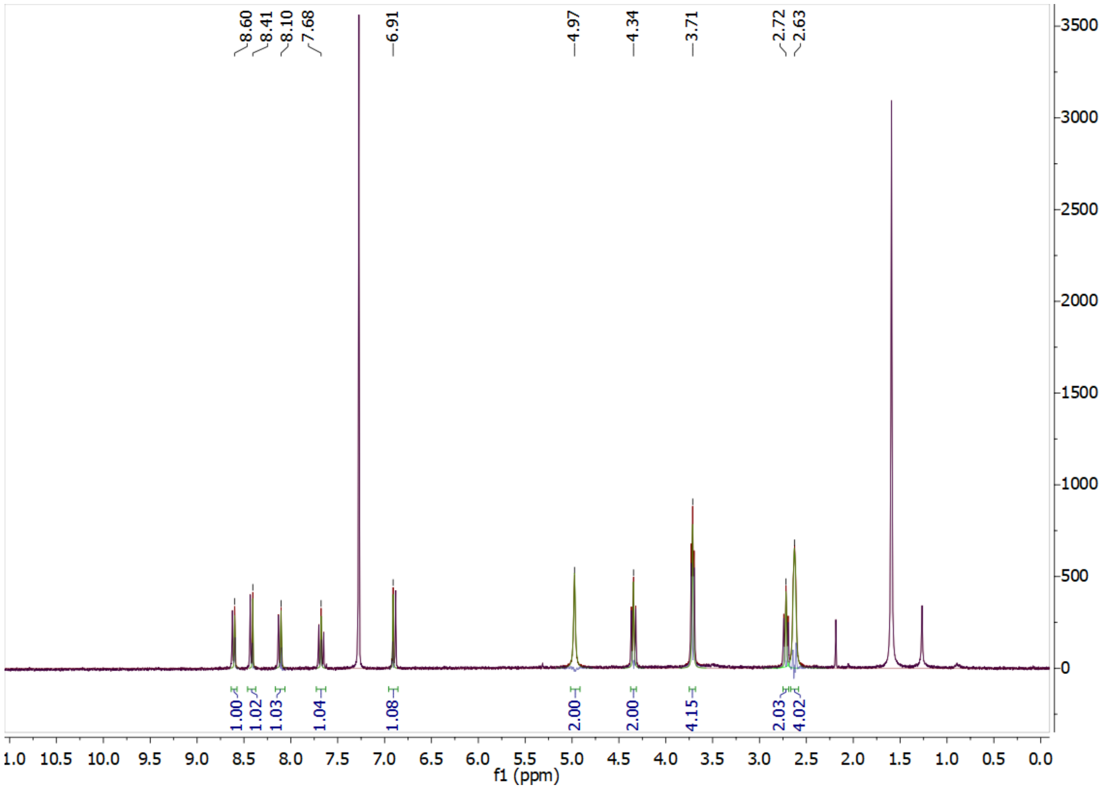

Figure S2. ^{1}H NMR spectrum (300 MHz, $CDCl_3$) of **LysoH$_2$O$_2$**.

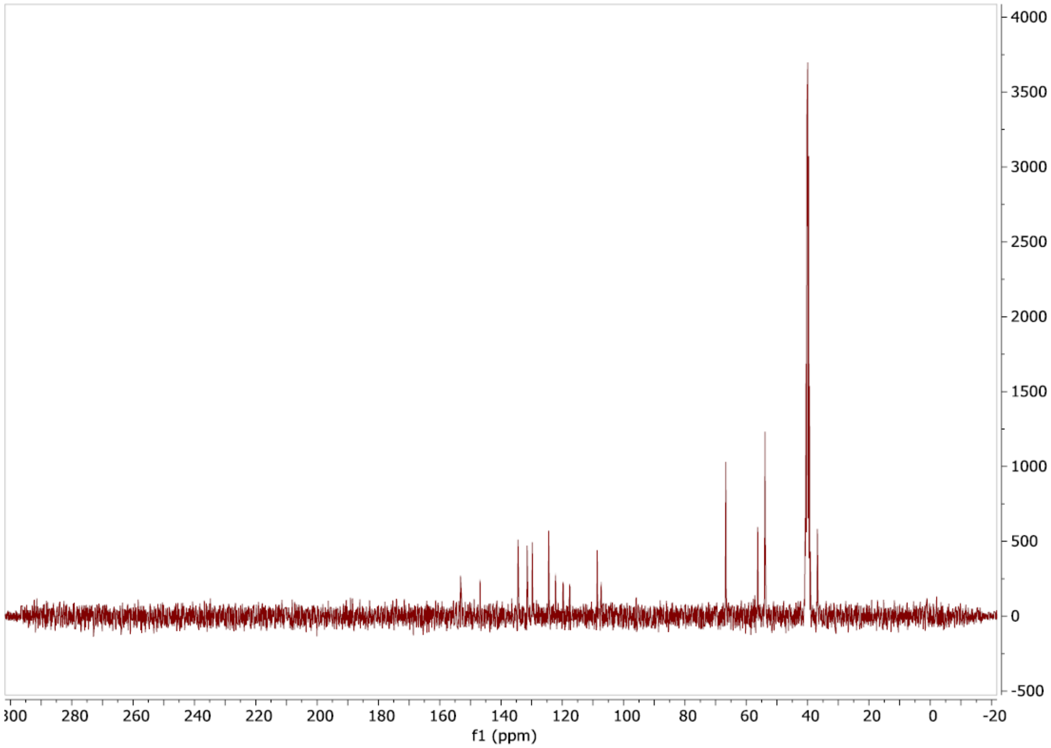

Figure S3. ^{13}C NMR spectrum (75 MHz, $CDCl_3$) of **LysoH$_2$O$_2$**.

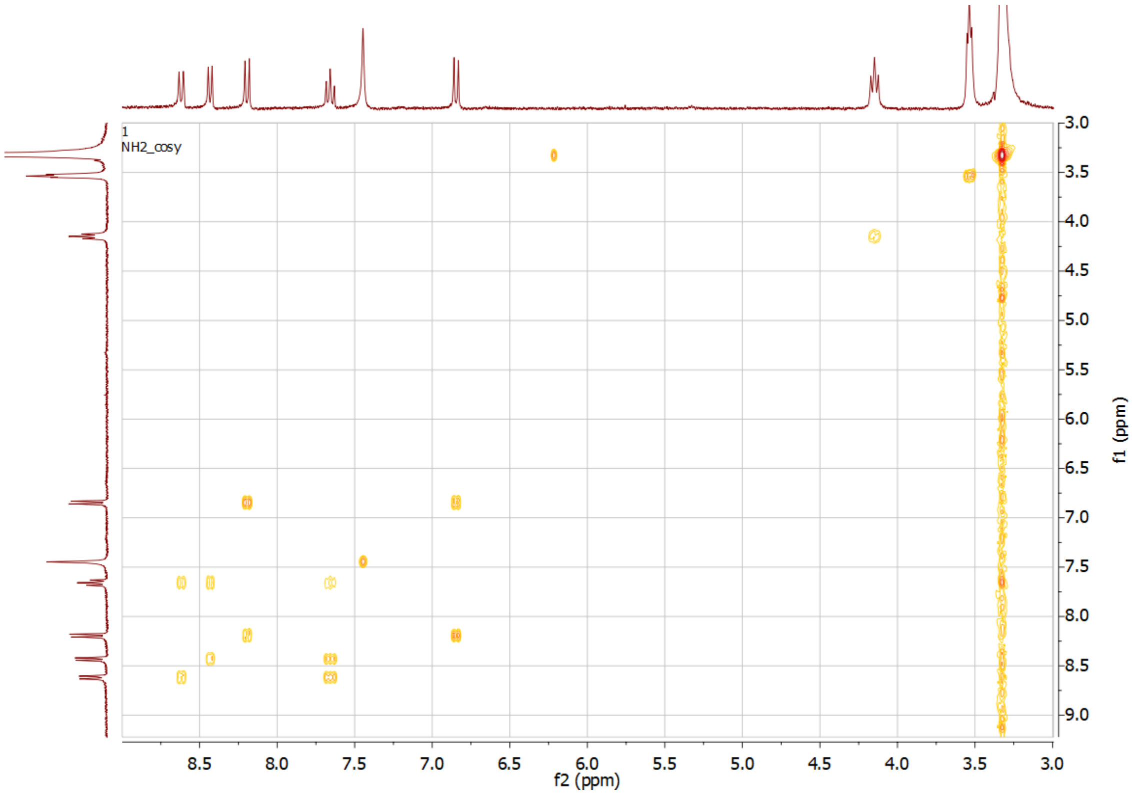

Figure S4. ${}^{1}H$-${}^{1}H$ COSY NMR spectrum (300 MHz, $CDCl_3$) of **LysoH_2O_2**.

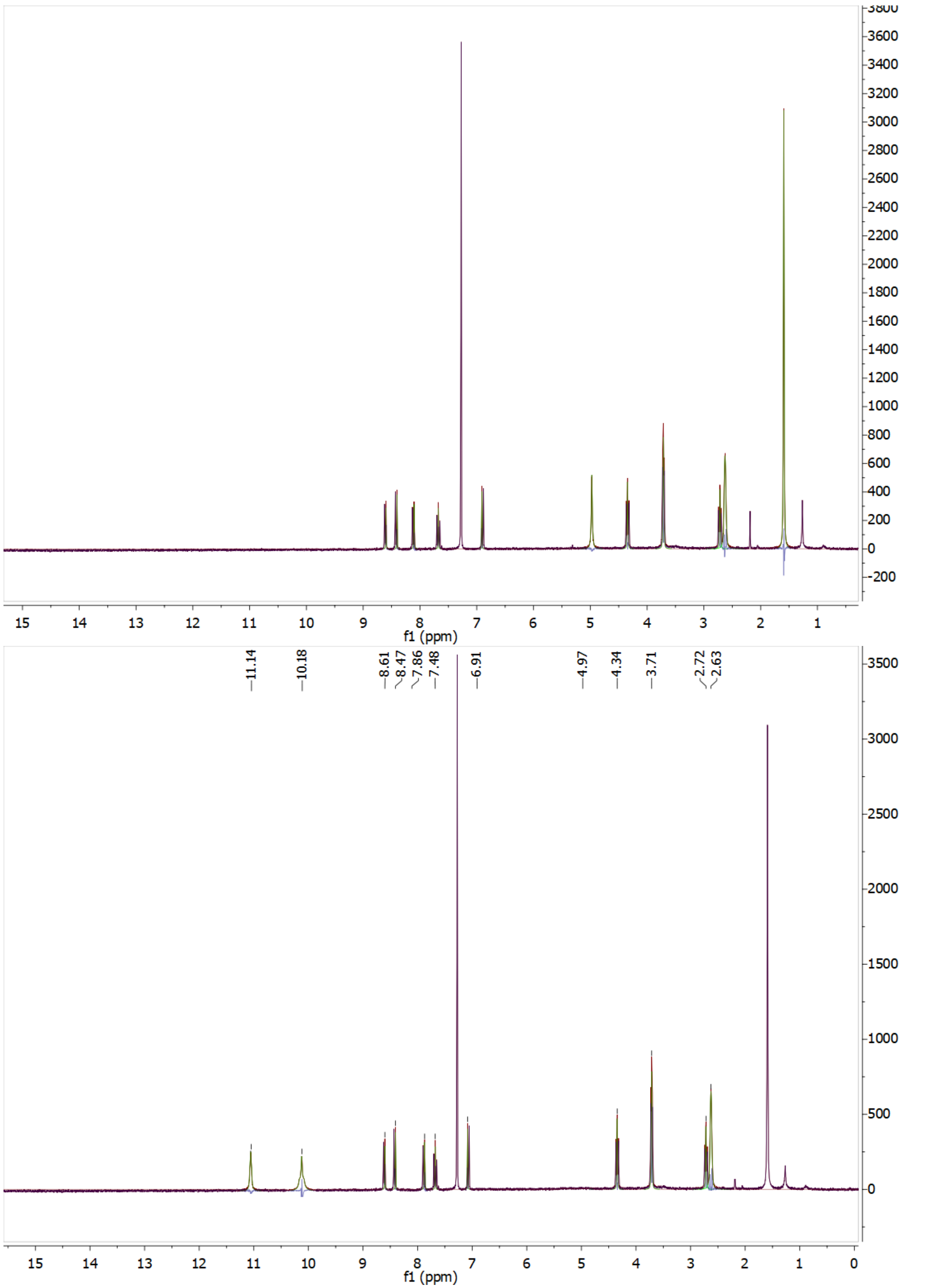

Figure S5. ^{1}H-NMR spectra (300 MHz, $CDCl_3$) of **LysoH_2O_2** (above) and after adding 1 equiv H_2O_2 14 % (below, δ: 10.15 (s, 1H, -OH signal) and 11.05 (s, 1H, -NH- signal).

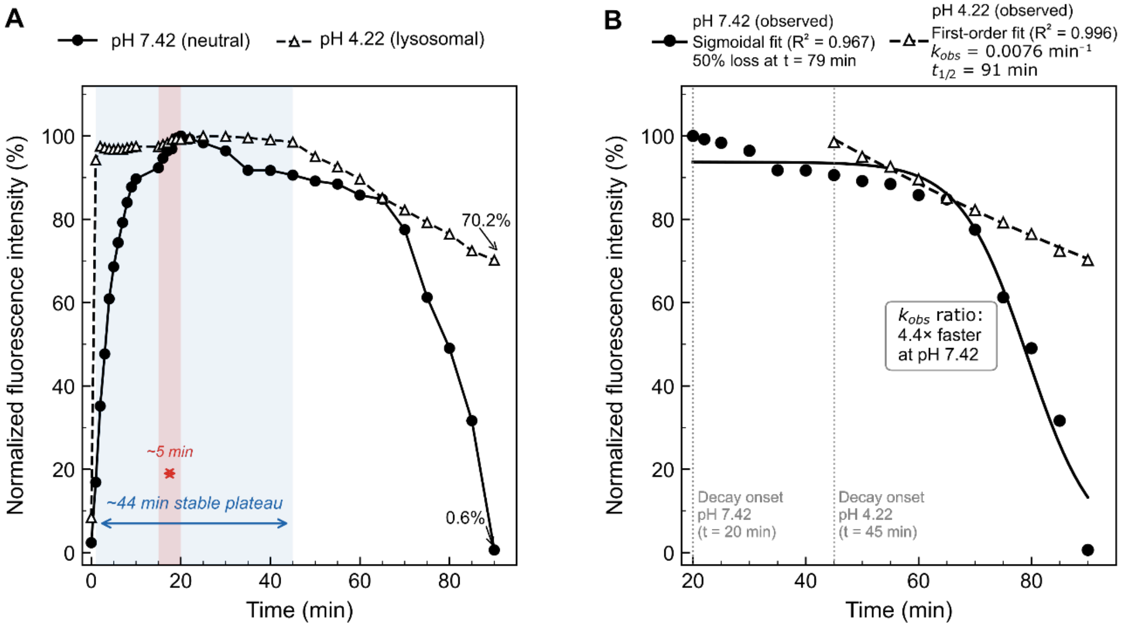

Figure S6. Extended fluorescence time-course profiles (λ_{exc} = 430 nm, λ_{em} = 550 nm) of **LysoH$_2$O$_2$** (40 µM) following activation by 2 equiv. H_2O_2 at pH 4.22 (5 mM HTAB micellar medium, open triangles, dashed line) and pH 7.42 (aqueous medium, filled circles, solid line), monitored over 90 minutes. (A) Full normalized profiles showing the rapid fluorescence turn-on upon H_2O_2 addition, the stable plateau phase at pH 4.22 (~44 min), and the contrasting immediate decay onset at pH 7.42. Shaded regions indicate the plateau windows at each pH. Retention values at t = 90 min are indicated. (B) Decay phase analysis with kinetic fits: sigmoidal decay fit for pH 7.42 (50% signal loss at t = 79 min, R^2 = 0.967) and pseudo-first-order decay fit for pH 4.22 (k_{obs} = 0.0076 min^{-1}, $t_{1/2}$ = 91 min from decay onset at t = 45 min, R^2 = 0.996). Vertical dotted lines indicate decay onset times. The 4.4-fold difference in decay rate constants between pH 7.42 and pH 4.22 quantifies the efficiency of the lysosomal acid-protection mechanism. Data are normalized to the maximum fluorescence intensity observed at each pH condition.

Reporte de Análisis Cualitativo
LANCIC IQ

**Data Filename**	MNH2.d	**Sample Name**	MNH2
**Sample Type**	Sample	**Position**	P1-D5
**Instrument Name**	LC QTOF-LANCIC	**User Name**	
**Acq Method**	Ine directa pos (ACN).m	**Acquired Time**	2/10/2025 1:28:09 PM
**IRM Calibration Status**	Success	**DA Method**	Everardo ESI MS.m
**Comment**			

**Sample Group**		**Info.**	
**Stream Name**	LC 1	**Acquisition SW**	6200 series TOF/6500 series
		**Version**	Q-TOF B.06.01 (B6172 SP1)

User Spectra

Fragmentor Voltage	Collision Energy	Ionization Mode
150	0	ESI

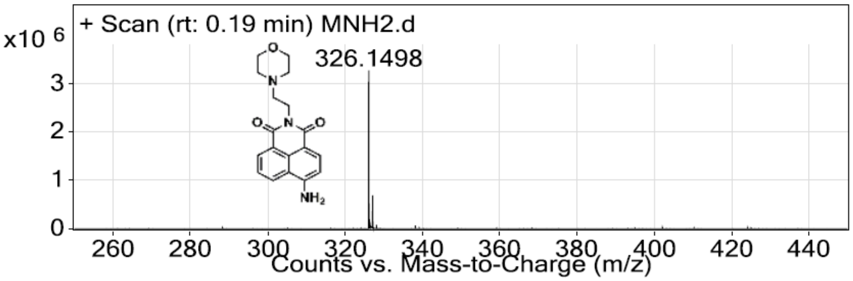

Peak List

m/z	z	Abund
326.1498	1	3295260.56
326.2962		207846.56
327.1529	1	710666.12

Figure S7. HRMS (ESI-TOF+) spectra of **LysoH_2O_2** (m/z: Calculated for $C_{18}H_{20}N_3O_3$ $[M+H]^+$: 326.14, found: 326.1498).

**Data Filename**	MNHOH.d	**Sample Name**	MNHOH
**Sample Type**	Sample	**Position**	P1-F2
**Instrument Name**	LC QTOF-LANCIC	**User Name**	
**Acq Method**	Ine directa pos (ACN).m	**Acquired Time**	2/10/2025 2:25:16 PM
**IRM Calibration Status**	Success	**DA Method**	Everardo ESI MS.m
**Comment**			

**Sample Group**		**Info.**	
**Stream Name**	LC 1	**Acquisition SW Version**	6200 series TOF/6500 series Q-TOF B.06.01 (B6172 SP1)

User Spectra

Fragmentor Voltage	Collision Energy	Ionization Mode
150	0	ESI

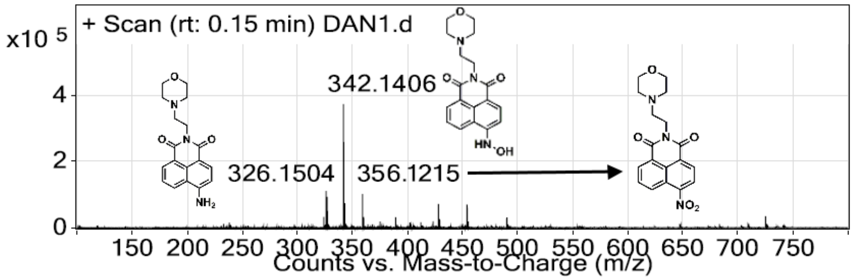

Peak List

m/z	z	Abund
326.1504	1	111884.59
327.153	1	93804.62
342.1406	1	385306.57
356.1215	1	92353.7
428.1815	1	70188.59
454.1722	1	70014.18
725.2416	1	35472.96

Figure S8. HRMS (ESI-TOF+) spectra of **LysoH$_2$O$_2$** after adding 1 equiv H_2O_2 14 % (m/z: Calculated for **hydroxylamine** derivative $C_{18}H_{20}N_3O_4$ [M+H]$^+$: 342.14, found: 342.1406). Also, the **nitro-oxidized** derivative was seen, m/z: $C_{18}H_{18}N_3O_5$ [M+H]$^+$: 356.12, found: 356.1215).

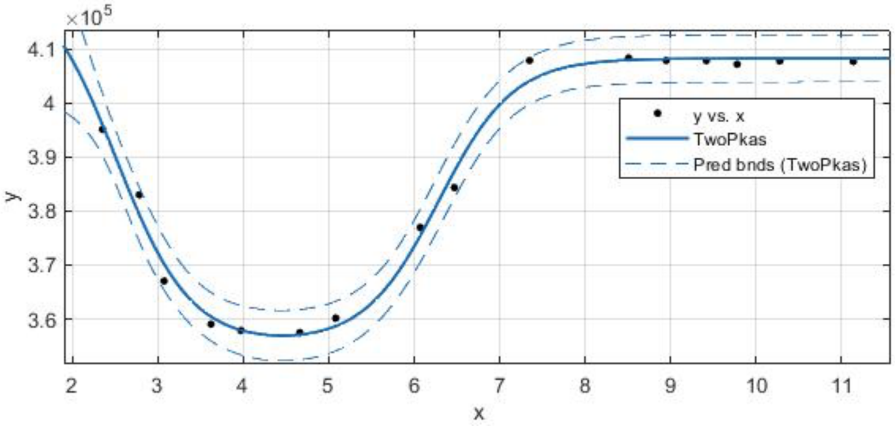

Coefficients with extrapolation (with 95% confidence bounds):

P1 = 4.083e+05 (4.063e+05, 4.102e+05)

P2 = 3.554e+05 (3.522e+05, 3.586e+05)

P3 = pKa's = (**2.509**, **6.285**)

P4 = 4.241e+05 (3.962e+05, 4.519e+05)

Goodness of fit:

SSE: 5.641e+07

R-square: 0.9918

Adjusted R-square: 0.9888

RMSE: 2264

Figure S9. Acid-base profiles and pK_a analysis for **LysoH$_2$O$_2$**

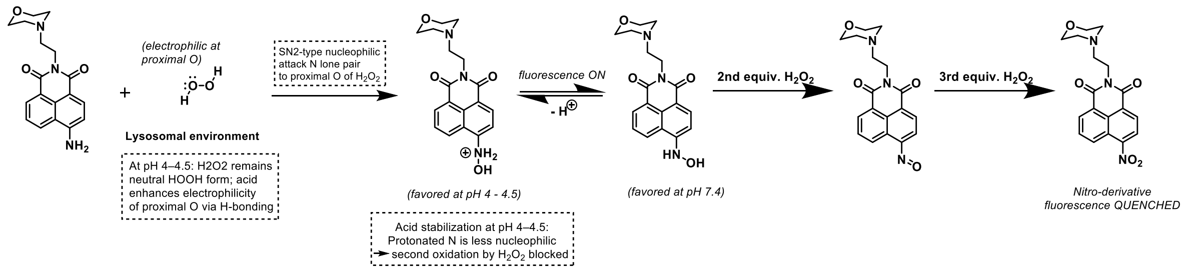

Figure S10. H_2O_2 oxidation mechanism of **LysoH$_2$O$_2$**. In the absence of enzymatic catalysis, the reaction of **LysoH$_2$O$_2$** with H_2O_2 in the acidic lysosomal environment (pH 4–4.5) proceeds via direct electrophilic oxygen transfer from H_2O_2 to the aromatic amine nitrogen, following a nucleophilic SN2-type mechanism at the peroxide oxygen, with water as the leaving group.[E. J. Behrman and J. O. Edwards, in *Progress in Physical Organic Chemistry*, ed. A. Streitwieser and R. W. Taft, Wiley, New York, 1967, **4**, 93–155.] The protonated hydroxylamine intermediate $[Ar–NH_2–OH]^+$ is stabilized at pH 4–4.5 through protonation of the nitrogen, which diminishes its nucleophilicity and prevents a second oxidative event. At physiological pH (7.42), the free base form Ar–NH–OH is a more reactive nucleophile, facilitating overoxidation to the nitroso and subsequently nitro derivatives with complete fluorescence quenching.[B. G. Gowenlock and G. B. Richter-Addo, *Chem. Rev.*, 2004, **104**, 3315–3340.] This pH-gated selectivity constitutes the core operating principle of the lysosomal protection mechanism in **LysoH$_2$O$_2$**.

Table S1. Photophysical parameters of **$LysoH_2O_2$** and its hydroxylamine product in 5 mM HTAB micellar medium at pH 4.22.

Parameter	$LysoH_2O_2$ (probe)	Hydroxylamine product
λ_{abs} (nm)	480	480
λ_{em} (nm)	480–500	550
ε ($M^{-1}cm^{-1}$)	6,550	6,890
Φf [2]	0.008	0.21
Turn-on factor (Φ/Φ_0)	—	26.3
Medium	5 mM HTAB, pH 4.22	5 mM HTAB, pH 4.22

[1] *$\lambda abs = \lambda em$ = 480 nm for both the probe and the hydroxylamine product.*

[2] *Fluorescence quantum yields (Φf) determined by the relative method using fluorescein in 0.1 M NaOH (Φ_{ref} = 0.95) as the reference standard. All measurements performed at room temperature (25 °C).*

Experimental procedure: fluorescence quantum yield determination

Fluorescence quantum yields (Φf) were determined using the relative method of Würth *et al.*,[1] with fluorescein in 0.1 M NaOH (Φ_{ref} = 0.95) as the reference standard. This method requires recording the UV-Vis absorption and fluorescence emission spectra of both the reference and the sample solutions at multiple dilutions under identical instrumental conditions.

Sample preparation. Stock solutions of **$LysoH_2O_2$** and its hydroxylamine product were prepared in 5 mM HTAB micellar medium at pH 4.22 (acetate buffer). The hydroxylamine product was generated in situ by treatment of **$LysoH_2O_2$** (40 µM) with 5 equivalents of H_2O_2 at pH 4.22, followed by confirmation of complete activation by fluorescence spectroscopy prior to quantum yield measurements. Working solutions were prepared at five different concentrations spanning a range of absorbance values between 0.01 and 0.10 at the excitation wavelength (λ_{exc} = 480 nm) to minimize inner filter effects.

Spectroscopic measurements. UV-Vis absorption spectra were recorded on a Thermo Scientific Evolution diode array spectrophotometer. Fluorescence emission spectra were recorded on an Edinburgh Instruments FS500 spectrofluorometer (or Cary Eclipse fluorimeter) using an excitation wavelength of λ_{exc} = 480 nm, with matched slit widths for both excitation and emission monochromators. All measurements were performed at room temperature (25 °C) under identical instrumental settings. The fluorescence spectra were corrected for instrument response.

Calculation. The integrated fluorescence intensity was plotted against the corresponding absorbance value at λ_{exc} *for each solution. The quantum yield of the sample (Φf)* was calculated according to the following equation:

$$\Phi f = \Phi_{ref} \times (m_{sample} / m_{ref}) \times (n_{sample}^2 / n_{ref}^2)$$

where Φ_{ref} is the quantum yield of the reference standard (fluorescein, Φ_{ref} = 0.95 in 0.1 M NaOH); m_{sample} and m_{ref} are the slopes of the integrated fluorescence intensity *versus* absorbance plots for the sample and reference, respectively; and n_{sample} and n_{ref} are the refractive indices of the solvents used for the sample and reference solutions, respectively (n = 1.333 for aqueous media at 25 °C; refractive index correction factor = 1 when both sample and reference are measured in aqueous solution).

Precautions. *All absorbance values were kept below 0.10 to avoid inner filter effects. Samples were freshly prepared prior to each measurement and protected from ambient light to prevent photobleaching. Each measurement was performed in triplicate and the reported* Φf values represent the mean of three independent determinations.

Reference: [1] Würth, C., Grabolle, M., Pauli, J. et al. Relative and absolute determination of fluorescence quantum yields of transparent samples. Nat Protoc 8, 1535–1550 (2013). https://doi.org/10.1038/nprot.2013.087

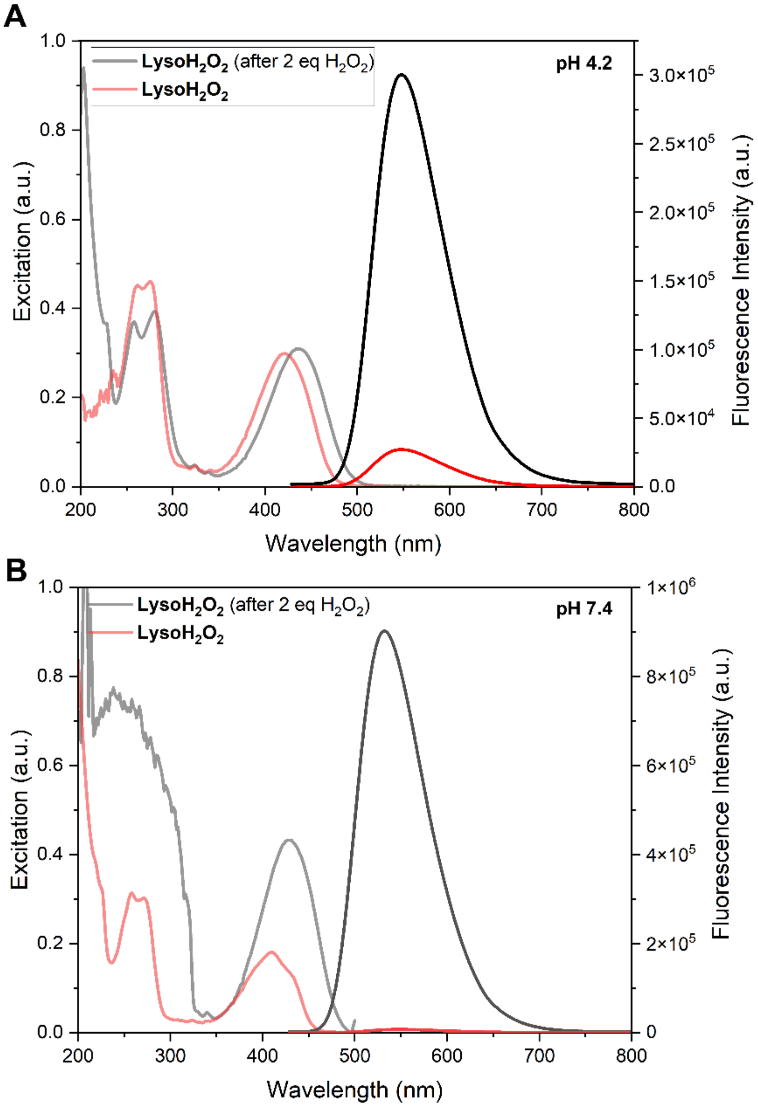

Figure S11. Fluorescence excitation (λ_{em} = 550 nm) and emission profiles (λ_{exc} = 440 nm) of 40 μM **LysoH$_2$O$_2$** probe before (red lines) and after (black lines) exposure to 2 equiv. H_2O_2 at pH 4.22 (A) and pH 7.42 (B).

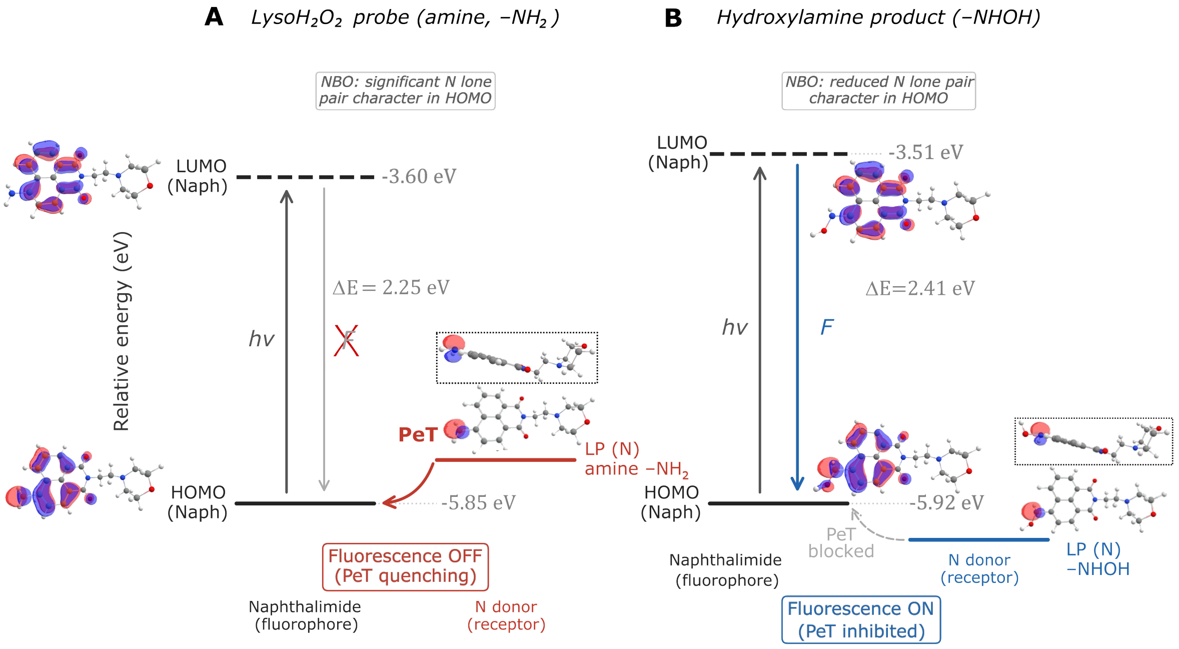

Figure S12. Frontier orbital energy alignment diagram comparing **LysoH$_2$O$_2$** (left) and its hydroxylamine product (right), computed at the PBE/6-31G(d,p) level of theory, with NBO orbital representations. Energy levels are given relative to vacuum. In the intact probe, the naphthalimide HOMO and LUMO are located at −5.85 eV and −3.60 eV, respectively (ΔE = 2.25 eV). The nitrogen lone pair (LP) of the amine –NH$_2$ group lies above the naphthalimide HOMO, enabling photoinduced electron transfer (PeT) from the donor to the photoexcited fluorophore, which quenches fluorescence (OFF state). Upon H_2O_2-mediated oxidation to the hydroxylamine product, the naphthalimide HOMO and LUMO shift to −5.92 eV and −3.51 eV, respectively (ΔE = 2.41 eV, ΔΔE = +0.16 eV). The LP of the –NHOH group is lowered below the naphthalimide HOMO, blocking PeT and restoring fluorescence emission (ON state). NBO analysis confirms significant nitrogen lone pair character in the HOMO of the probe (inset, left) and a marked reduction thereof in the hydroxylamine product (inset, right), consistent with PeT inhibition upon oxidation. LP energies are qualitative, derived from isolated fragment NBO calculations of the free amine and hydroxylamine groups. Due to direct conjugation of the amine nitrogen into the naphthalimide π-system, frontier molecular orbitals are delocalized across both donor and acceptor units; the diagram is therefore presented as a qualitative mechanistic representation consistent with established models for 4-aminonaphthalimide-based fluorescent sensors.
